# A TILLING Platform for Functional Genomics in *Brachypodium distachyon*

**DOI:** 10.1371/journal.pone.0065503

**Published:** 2013-06-19

**Authors:** Marion Dalmais, Sébastien Antelme, Séverine Ho-Yue-Kuang, Yin Wang, Olivier Darracq, Madeleine Bouvier d’Yvoire, Laurent Cézard, Frédéric Légée, Eddy Blondet, Nicolas Oria, Christelle Troadec, Véronique Brunaud, Lise Jouanin, Herman Höfte, Abdelafid Bendahmane, Catherine Lapierre, Richard Sibout

**Affiliations:** 1 URGV, Unité de Recherche en Génomique Végétale, Université d’Evry Val d’Essonne, INRA, Evry, France; 2 Institut National de la Recherche Agronomique, Institut Jean-Pierre Bourgin, Saclay Plant Sciences, Versailles, France; 3 AgroParisTech, Institut Jean-Pierre Bourgin, Saclay Plant Sciences, Versailles, France; East Carolina University, United States of America

## Abstract

The new model plant for temperate grasses, *Brachypodium distachyon* offers great potential as a tool for functional genomics. We have established a sodium azide-induced mutant collection and a TILLING platform, called “BRACHYTIL”, for the inbred line Bd21-3. The TILLING collection consists of DNA isolated from 5530 different families. Phenotypes were reported and organized in a phenotypic tree that is freely available online. The tilling platform was validated by the isolation of mutants for seven genes belonging to multigene families of the lignin biosynthesis pathway. In particular, a large allelic series for *BdCOMT6*, a caffeic acid O-methyl transferase was identified. Some mutants show lower lignin content when compared to wild-type plants as well as a typical decrease of syringyl units, a hallmark of COMT-deficient plants. The mutation rate was estimated at one mutation per 396 kb, or an average of 680 mutations per line. The collection was also used to assess the Genetically Effective Cell Number that was shown to be at least equal to 4 cells in *Brachypodium distachyon*. The mutant population and the TILLING platform should greatly facilitate functional genomics approaches in this model organism.

## Introduction

*Brachypodium distachyon* is a valuable model plant for economically important temperate grasses such as wheat, barley and oat [1]. Attractive features are the small size, simple culture conditions and the small (272 Mb), diploid (2n = 10) and fully sequenced genome [2]. *B. distachyon* is currently being used as a model for the study of domestication in grasses [1], [2], [3], [4], [5], plant pathogen interactions, root and culm development, biomass production and cell wall biosynthesis. Important progress has been made in the development of efficient transformation protocols [6], [7], [8] and sequence-indexed T-DNA insertion collections [9]: the BrachyTAG collection at the John Innes Centre (5000 lines) ([10], http://www.brachytag.org/) and the USDA Brachypodium Genome Resources collection (8491 lines) [11]. To extend the panel of resources for functional genomics in this species, we have developed a mutagenized population and a TILLING (Targeting Induced Local Lesion IN Genome) platform. Chemically-induced mutants are complementary to insertion mutants in several aspects: (i) mutation rates are higher and hence screens can be done on smaller mutant populations, (ii) single base pair changes, as opposed to insertion mutants, more likely yield allelic series of partial loss-of-function, conditional or gain-of-function mutants and (iii) somaclonal variation is avoided in the absence of an *in vitro* culture step that is required for T-DNA-induced mutagenesis [12], [13]. An efficient method to identify new alleles for target genes is TILLING. This method developed a decade ago has been successfully applied to many plant species [14], [15]. In the TILLING method, seeds are mutagenized, the resulting M1 plants are self-fertilized, and the M2 generation of individuals is used to prepare DNA samples for mutational screening, while seeds of the M3 families can be stored and distributed. DNA samples are pooled and subjected to gene-specific PCR. The amplification products are incubated with an endonuclease that preferentially cleaves mismatches in heteroduplexes between wild type and mutant. Upon detection of a mutation in a pool, the individual DNA samples are similarly screened to identify the plant carrying the mutation. TILLING populations have been established for grasses including wheat, sorghum, barley, rice and oat [15], [16], [17], [18], [19], [20], [21], [22], [23] but to our knowledge not yet for *Brachypodium distachyon*. Here we report on the development of a mutagenized population and a TILLING platform and demonstrate the efficiency of the TILLING method with the identification of a series of allelic mutants for an *O*-methyl transferase, involved in the lignification of the internodes.

## Materials and Methods

### Plant Material and Growth Conditions

*Brachypodium distachyon* (L.) Beauv. inbred line Bd21-3 was kindly provided by John Vogel. Bd21-3 seeds were grown in a greenhouse under long-day conditions (18 h light, 400 watt sodium lamps). Day and night temperatures were 23°C and 18°C, respectively. The relative humidity was about 60%. Plants were grown in soil (one-liter pots) and watered twice a week.

### Chemical Mutagenesis

For the production of mutants, dry seeds were pre-soaked in distilled water for 2 h. Portions of 5000 seeds were then suspended in 200 mL of fresh sodium azide (NaN_3_) solution diluted in phosphate buffer (0.1 M, pH 3) for 2 h under the hood and with gentle shaking. The seeds were washed 3 times in water for 1 h and then kept at +4°C for 72 h before sowing in pots. For establishment of the kill curve, 500 seeds were mutagenized with 0.5, 1, 1.5, 3 or 10 mM NaN_3_.

### Genomic DNA Extraction and Pooling

Four M2 plants per family were grown for one month in a greenhouse. DNA was extracted from 3 cm-long portions of the median foliar part. The collected samples were pooled and placed in 96-well plates containing 2 steel beads per well. Samples were lyophilized and ground using a bead mill. Genomic DNAs were isolated using the DNeasy 96 Plant Kit (Qiagen, Hilden, Germany). All genomic DNAs were both quantified on a 1% agarose gel with λ DNA (Invitrogen, Carlsbad, CA, USA) as a concentration reference using a NanoDrop spectrophotometer 2000 c (Thermo Fisher Scientific, MA, USA). DNA concentration was normalized to 6 ng.µL^−1^ and pooled eightfold in a 96-well format.

### PCR Amplification and Detection of Mutations

DNA amplification is based on nested-PCR. The first PCR amplification is a standard PCR reaction with target-specific primers and 10 ng of *Brachypodium* genomic DNA. One µl of the first PCR product served as a template for the second nested PCR amplification, with a combination of specific primers carrying M13 tail and M13 universal primers, M13F700 (5′-CACGACGTTGTAAAACGAC-3′) and M13R800 (5′-GGATAACATTTCACACAGG-3′), labelled at the 5′end with infra-red dyes IRD700 and IRD800 (LI-COR®, Lincoln, Nebraska, USA) respectively. Mutation detection was carried out as described previously except for the second PCR. This PCR was carried out using 0.05 µM of specific primers carrying M13 tail and 0.1 µM of M13 universal primers. The identity of the mutations was determined by sequencing.

### Sequence Analysis Tools

The CODDLE software (Codons Optimized to Discover Deleterious Lesions, http://www.proweb.org/coddle/) was used to identify regions of the target gene in which G/C to A/T transitions are most likely to result in deleterious effects on the protein. The PARSESNP software (Project Aligned Related Sequences and Evaluate SNPs, http://www.proweb.org/parsesnp/) was used to illustrate the distribution of mutations within the gene and to indicate the nature of each single mutation. The SIFT software (Sorting Intolerant from Tolerant, http://sift.jcvi.org/www/SIFT_seq_submit2.html) was used to predict the impact of the mutation on the protein. Multiple sequence alignment of full-length protein sequences was performed with ClustalW software (http://www.ebi.ac.uk/Tools/clustalw2).

### Phylogenetic Tree and 3D-structure Prediction

Protein sequences of COMT from several vascular plants were identified by protein blast (http://www.ncbi.nlm.nih.gov/and http://www.greenphyl.org) and aligned with Clustawl2 (pairwise alignment) at (http://www.ebi.ac.uk/Tools/msa/clustalw2). Phylogenetic analysis was performed with TreeTop (http://www.genebee.msu.su/services/phtree_reduced.html). Cluster algorithm was used and 100 bootstrap were done. Final construction of tree was made with TREX (Treeview Newick Viewer option) at http://www.trex.uqam.ca/. Bootstrap values are expressed in percentages and placed at nodes of the trees. The 3D-structure of the BdCOMT6 protein was predicted from the crystallized ryegrass enzyme [24] with the molecular modeling program Geno3D (http://geno3d-pbil.ibcp.fr/) and UCSF Chimera software (http://www.cgl.ucsf.edu/chimera/).

### Lignin Analyses

All reagents and solvents were high-quality grade commercial reagents employed without further purification. Dried mature stems (3 month old) from wild-type (WT) plants or from azygous (mutagenized plants seggregating the *WT* allele for the studied locus) or homozygous mutant plants were ground to 0.5 mm before exhaustive extraction with water, then ethanol in a Soxhlet apparatus. The lignin content was measured on the recovered extractive-free samples and by the Klason method [25]. Lignin structure was evaluated by thioacidolysis performed from 10 to 20 mg of extractive-free sample put together with 0.5 mg of C21 internal standard in 10 mL of reagent (dioxane/ethanethiol 9/1 V/V, containing 0.2 M BF_3_ etherate) and incubated at 100°C for 4 h. After the reaction, the lignin-derived monomers were extracted with methylene chloride as previously described [26], the combined organic extracts were concentrated to about 2 mL and then 4 µL of the sample were silylated by 100 µL BSTFA and 5 µL pyridine before injection onto a DB1 supelco capillary columns (carrier gas helium, constant flow rate 1 mL/min) operating from 40 to 180°C at +30°C/min, then 180 to 260°C at +2°C/min and combined with an ion trap mass spectrometer (Varian Saturn2100) operating in the electron impact mode (70 eV), with ions detected on the 50–600 m/z range. The surface area of the *p*-hydroxyphenyl (H), guaiacyl (G), syringyl (S) and 5-hydroxyguaiacyl (5-OH G) monomers were measured on specific ion chromatograms, at m/z 239, 269, 299, and 357 respectively.

## Results and Discussion

### Production of a Mutagenized Collection

**Figure 1:**
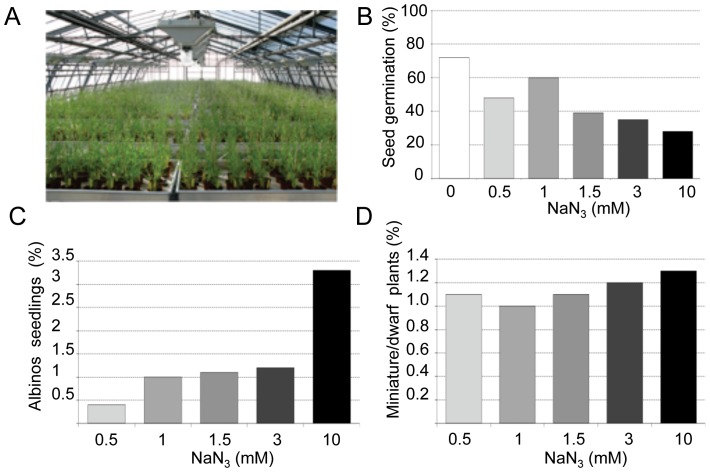
Production of Brachypodium mutants. A. 3000 M1 mutagenized individual plants growing in greenhouse. B. Percentage of seeds germinating after 2 hours imbibition in different NaN_3_ concentrations. C. Percentage of M2 albino seedlings observed according to NaN_3_ concentrations. D. Percentage of M2 dwarf/miniature plants observed according to NaN_3_ concentrations. In each experiment (B–D) 100 seeds per concentration were sowed.

When grown under long-day conditions in our greenhouses (18 h light, Figure 1A), the life cycle was about 12 weeks. Sodium azide (NaN_3_) was used to mutagenize *B. distachyon* Bd21-3 seeds. This compound is commonly used for mutagenesis of grasses [15] and the frequency of chromosome breakage is relatively low. To establish a dose-response kill curve, we determined the fraction of M1 seeds that germinated after imbibition with increasing NaN_3_ concentrations (Figure 1B). The lowest germination frequency was 28% for 10 mM NaN_3_. The frequency of albino M2 seedlings (Figure 1C) and miniature/dwarf plants (Figure 1D) also showed a dose-dependent increase with for instance a maximum frequency of 3.3% and 1.3% albinos respectively for 10 mM and 3 mM NaN_3_ (Figure 1D). We concluded that 3 mM and 10 mM NaN_3_ provided a good compromise combining high mutation rate and sufficient germination frequency. These concentrations were used to generate the mutagenized population of 5530 individual families.

### Plant Phenotyping

**Figure 2:**
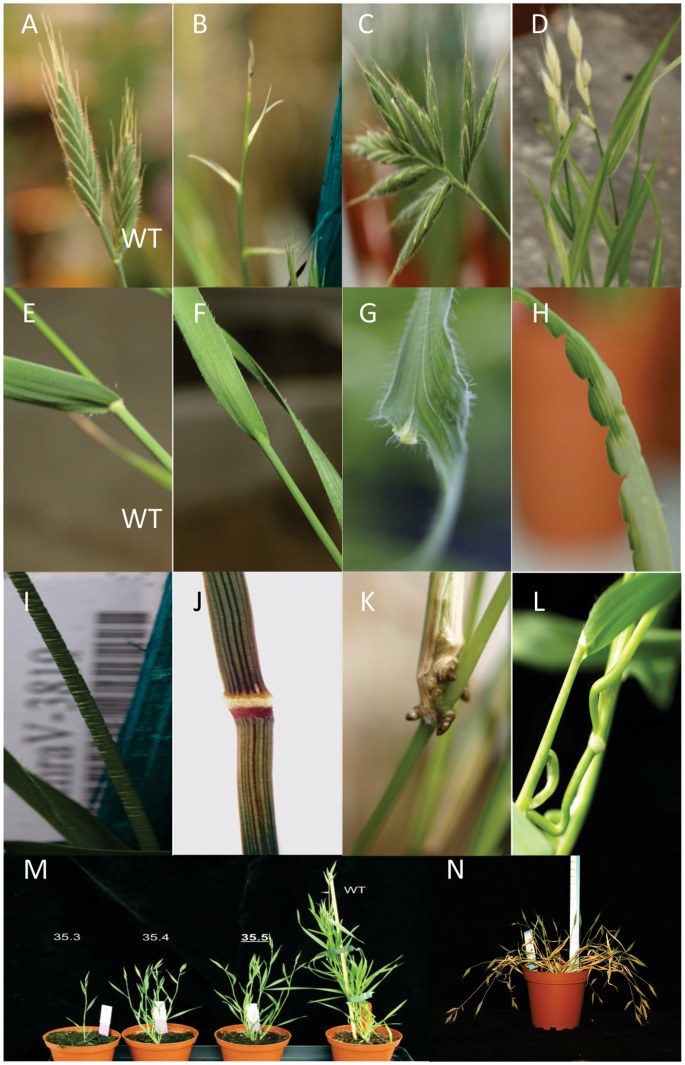
Examples of phenotypes detected in the Brachypodium mutant collection. A. Spikelets of wild-type plants. B–D Aborted or abnormal spikelets in mutants. E. Ligule of wild-type plant. F. Abnormal ligule in mutant. G, H. Mis-shaped leaves in mutants. I. Crumple stem in mutant. J. Coloured stem in mutant. K. adventitious roots in mutant. L. Curved stems in mutant. M. Segregant phenotype for tiller formation in M2 plants compared to WT (right). N. Floppy stems in mutant.

The phenotypes (shape, color and size) of a subset of mutants were recorded, using an ontology developed for sorghum [27], at three developmental stages: Germination/seedling stage, vegetative stage (before flowering) and reproductive stage (after flowering). Phenotypes were organized in a phenotypic tree available at http://urgv.evry.inra.fr/UTILLdb. Figure 2 and Table 1 illustrate the most striking phenotypes of greenhouse-grown mutants.

**Table 1:** Types of phenotypes observed on 565 phenotyped lines (3360 plants).

Major category	Phenotype sub-category^a^	Number of families affected	% of families affected
Dead	Not emerged or early death	340	
Plant devlopment	Delay Plant development	87	15.4
Plantlet	Albino	20	3.5
Leaf	Size	129	22.8
	Color	121	21.4
	Appearance	125	22.1
	Shape and arrangements	9	1.6
Stem	Shape	112	19.8
	Color	19	3.3
Node	Shape	1	0.2
Spike	Flowering time	52	9.2
	Spike organisation	62	11
	Reproductive organs	15	2.6
	Spike color	62	11
Plant architecture	Architecture	394	69.7
	Height and appearance	146	25.8
	Tillers	70	12.4
	Branching type	47	8.3

a phenotypes are available at http://urgv.evry.inra.fr/UTILLdb.

### TILLING Genes Involved in Lignin Biosynthesis

*B. distachyon* is increasingly used as a model for bioenergy grasses [28], [29] Lignins are the main obstacle in the enzymatic conversion of cellulose into fermentable sugars. We therefore chose to screen the mutant collection for genes involved in lignin biosynthesis as a validation of the tilling method [29]. We focused on seven genes belonging to multigene families that are potentially involved in monolignol biosynthesis (Table 2): one caffeic acid *O*-methyltransferase gene (*Bradi3g16530*), two laccase genes (*Bradi1g74320*, *Bradi1g66720*), one *4*-coumarate:coenzyme A ligase gene (*Bradi3g05750*) and three cytochrome P450 genes (*Bradi2g31510*, B*radi2g53470*, *Bradi3g43160*).

**Table 2:** Tilled genes and mutation frequency in the mutant collection with 5530 M2 families screened.

Target names	Accession number	Amplicon size (bp)	GC content (%)	Identified mutants	Mutation frequency
COMT	Bradi3g16530	1245	65	23	1/300 kb
Laccase	Bradi1g74320	2372	43	17	1/771 kb
Laccase	Bradi1g66720	2607	51	18	1/801 kb
4CL	Bradi3g05750	735	68	17	1/239 kb
C4H	Bradi2g31510	643	65	14	1/254 kb
C4H	Bradi2g53470	728	63	12	1/335 kb
C4H	Bradi3g43160	829	61	27	1/170 kb
TOTAL		9159	59	128	1/396 kb

We identified a total of 128 mutations. The nucleotide changes induced by NaN_3_ are mainly G/A and C/T substitutions similar to those induced by ethyl methane sulfonate (EMS) for instance [30], [31]. Only 9% other mutations were detected (Table 3). We identified 63% non-synonymous mutations (including 53% and 5% inducing amino acid changes or stop codons, respectively). One line showed a mutation in a splicing region. Extrapolating from this small set of genes, we calculated an average mutation rate of one in around 400 kb (Table 2) or 700 mutations per genome for a genome size of 272 Mb [2], This frequency of induced mutations is similar to that found in EMS-mutagenized rice (1/294 kb, [23]) or in barley treated with NaN_3_ (1/374 kb, [22]) or EMS (1/500 kb, [20]).

**Table 3:** Frequencies of induced mutations types in tilled gene-coding regions.

	Accession number	Missense^a^	Nonsense^b^	Splicing^c^	Silent^d^	Unusual nucleic transition^e^
COMT	Bradi3g16530	13	0	0	10	0
Laccase	Bradi1g74320	8	3	0	6	0
Laccase	Bradi1g66720	8	0	1	5	3
4CL	Bradi3g05750	13	0	0	4	4
C4H	Bradi2g31510	10	1	0	3	1
C4H	Bradi2g53470	6	1	0	4	1
C4H	Bradi3g43160	13	1	0	13	2
TOTAL		71	6	1	45	11
TOTAL (%)		57.7	4.8	0.8	36.7	8.6

a nucleic acid transition is a non-synonymous mutation and induce amino acid change in the translated protein.

b nucleic acid transition produces a stop codon and may induce a truncated protein.

c nucleic acid transition is located in splicing motif.

d nucleic acid transition induces a synonymous mutation and then no change in the translated protein.

e other nucleic acid transition than guanine to adenine and cytosine to thymine.

We also used our mutagenized population to estimate the ‘Genetically Effective Cell Number (GECN)’. This is the number of cells within the shoot meristem of the embryo that will contribute to the seed output. The GECN is usually estimated by determining the proportion of mutant seedlings in M2 families [32], [33]. We took advantage of our sequencing data on M2 families from individual M1 plants segregating mutations in 4 genes. In 584 lines from 30 different families (between 9 and 49 M2 independent lines were analyzed per family) we detected 38 homozygous mutations corresponding to a ratio of 1∶15.3 ( = 38/584). The 1∶15 ratio corresponds to a GECN of 4. This is higher than in Arabidopsis (GECN = 2) but in accordance with the number found in other grass species [33], [34].

### Identification of a Caffeic acid *O*-methyltransferase (*COMT*) Gene Potentially Involved in Internode Lignification

*B. distachyon* lignins, like those in other grasses, are mainly composed of guaiacyl (G) and syringyl (S) units, with low amounts of *p*-hydroxyphenyl (H) units [35] [36]. These H, G and S lignin units respectively originate from the three monolignols, namely *p*-coumaryl, coniferyl, and sinapyl alcohols that differ only in the degree of methoxylation of the phenolic ring [37]. The main role of the COMT involved in lignification is the methylation of 5-hydroxyconiferaldehyde to produce sinapaldehyde, which is reduced by another enzyme to sinapyl alcohol, the precursor of S lignin units. The COMT enzyme belongs to the S-Adenosyl methionine (SAM)-dependent *O*-methyltransferases. It is active as a homodimer and does not need any metal ion as cofactor. The hallmark of transgenic or mutant angiosperms with strongly repressed COMT activity is a reduction of the amount of S lignin units (or of the S/G ratio) together with the appearance of easily detectable amounts of 5-hydroxyguaiacyl (5-OH G), which is present only in trace amounts in the WT [38] [39], [40], [41], [42]. Another trait of some *COMT*-mutant grass lines, referred to as the brown-midrib mutants, is a lower lignin level resulting in a higher enzymatic degradability [43], and reduced levels of *p*-coumaric acid (CA) ester-linked to the cell walls [44].

**Figure 3:**
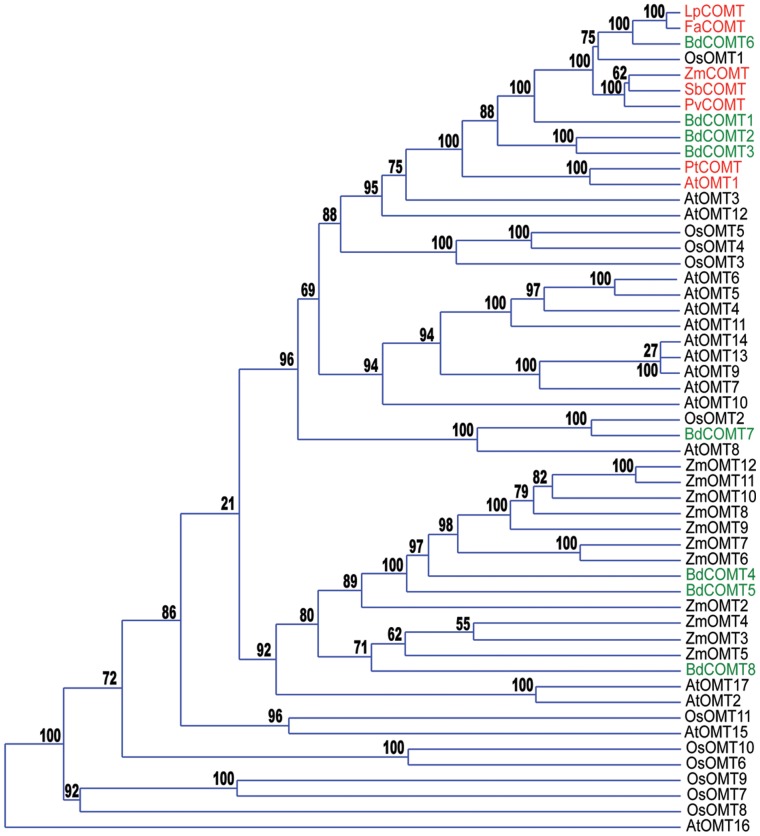
Phylogenetic analysis of putative COMT proteins. Phylogeny tree (phylogram) made with OMT proteins from Brachypodium (BdCOMT), rice (OsOMT), maize (ZmOMT) and Arabidopsis (AtOMT). The proteins known to be involved in lignification in ryegrass (LpCOMT), sorghum (SbCOMT), switchgrass (PvCOMT), fescue (FaCOMT) and poplar (PtCOMT) are included in the analysis and shown in red in the phylogram as well as Arabidopsis (AtOMT1) and Maize (ZmCOMT1) proteins. Brachypodium proteins (BdCOMT) are shown in green. Protein sequences are available in Information S1. Bootstrap values indicating the level of support for the displayed representation after re-sampling are shown on each node.

To unambiguously identify the *B. distachyon COMT* gene specifically involved in lignification, protein sequences of orthologs in several species were BLASTed onto the *B. distachyon* predicted proteome sequence. Eight proteins were identified: BdCOMT1 (Bradi1g14870), BdCOMT2 (Bradi2g02380), BdCOMT3 (Bradi2g02390), BdCOMT4 (Bradi2g19830), BdCOMT5 (Bradi2g19850), BdCOMT6 (Bradi3g16530), BdCOMT7 (Bradi3g55890), BdCOMT8 (Bradi4g20020). We performed a phylogenetic analysis with the most exhaustive list of encoded OMT proteins found in *Oryza sativa*, *Arabidopsis thaliana* and *Zea mays* genomes [45], [46], [47] (Figure 3, Information S1). In addition, we added to the phylogenetic analysis the protein sequences for which biological data (transgenics or mutants) support indisputably a role of the corresponding protein in lignification of several grass (maize, ryegrass, tall fescue, switchgrass, sorghum) and poplar [24], [39], [42], [48], [49], [50], [51]. *AtOMT1* was clearly identified by our group as a unique gene involved in sinapyl alcohol biosynthesis in Arabidopsis since the knockout mutant displays a lignin devoid of S units [52]. Therefore, AtOMT1 is a reference model for dicot COMT proteins as well as maize ZmCOMT1 (BM3) is a reference for grasses [49]. The phylogenetic analysis shows that both dicot proteins (AtOMT1, PtOMT) cluster together whereas four members of BdCOMTs (BdCOMT3, BdCOMT2, BdCOMT1 and BdCOMT6) are grouped with the genuine grass COMT proteins, BdCOMT6 being the closest ortholog (figure 3). Consequently, *BdCOMT6* was chosen for TILLING. It is worth noting, that despite no *comt* mutant was identified in rice to our knowledge this analysis suggests strongly that OsOMT1 (Os08g06100) is involved in monolignol formation.

### Mutations in *BdCOMT6* Affect the Lignification of Mature Stems

We identified 25 lines, corresponding to 22 different mutations, among which eleven missense mutations cause changes in the BdCOMT6 amino acid sequence (Table 4). No induced stop codon mutation were identified. A first analysis using the SIFT software (using 0.05 as significant threshold) indicated that the substitutions caused by the mutations in lines *Bd6840*, *Bd4688, Bd4604, Bd7480, Bd5139, Bd7549, Bd7391, Bd4142* and *Bd4927* may partially or totally disrupt the COMT activity. Among them, mutations in lines *Bd5139* and *Bd7549* were found redundant, as well as those in lines *Bd7480* and *Bd4604*, thereby reducing the number of mutations potentially affecting COMT activity to seven.

**Table 4:** Allelic series of mutations in *BdCOMT6* gene identified by TILLING.

Nucleic acid transition^a^	Amino acid substitution^b^	SIFT^c^	Family name
G37A	Asp13Asn	0.18	5645
G117A	Leu39Leu	–	5714
C498T	Asp166Asp	–	5588
G588A	Glu196Glu	–	8240
C600T	Tyr200Tyr	–	5827
G616A	Gly206Ser	0.01	6840
G638A	Gly213Asp	0.26	3380
G638A	Gly213Asp	0.26	211
G672A	Gly224Gly	–	3725
G708A	Gly236Gly	–	5338
G721A	Gly241Arg	0.02	4688
G737A	Gly246Asp	0	4604
G737A	Gly246Asp	0	7480
C762T	Pro254Pro	–	5115
G767A	Gly256Asp	0	5139
G767A	Gly256Asp	0	7549
C840T	Cys280Cys	–	5348
C854T	Pro285Lys	0.02	7391
G969A	Gly323Gly	–	3730
G976A	Glu326Lys	0	4142
G1013A	Glu338Asp	0.14	5200
G1063A	Ala355Thr	0.01	4927
G1071A	Glu357Glu	–	185

a Position of transition in mutants are relative to the starting ATG on the coding sequence.

b Position of substitution in mutants are relative to the starting methionine of the encoded protein.

c numbers are predictive score from the SIFT software (http://sift.bii.a-star.edu.sg/).

**Figure 4:**
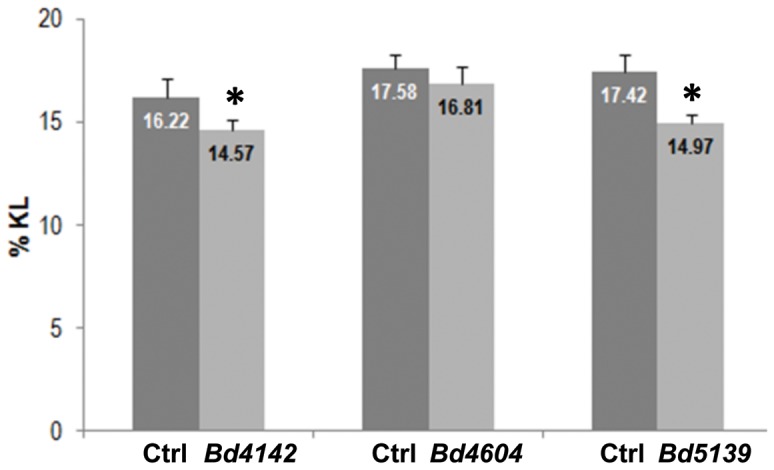
Klason lignin (KL) level of extractive-free mature stems of *Bd4142, Bd4604* and *Bd5139* lines as compared to control samples. Control samples are either wild-type (Bd21-3) or azygous plants grown together with the corresponding mutants. The KL level is expressed as weight percentage of the extractive-free sample. Data are means and SD from 4 or 5 plants analyzed per line. Asterisks indicate significant difference compared to the control (ANOVA value at P<0.05).

We next isolated homozygous lines for the all *comt* mutants, except for *Bd7480*. Indeed, this line failed to produce viable seeds at heterozygous stage. All genotyped plants were indistinguishable from WT plants when grown in the greenhouse except for homozygous *Bd211*, which was dwarfed. The dwarfism presumably is independent from the mutated COMT allele since *Bd3380*, which carries the same mutations was not dwarfed (Table 4). We therefore excluded *Bd211* from the subsequent analyses. We next studied the lignin composition in mature stems using thioacidolysis (reviewed in [53]). Thioacidolysis identifies H, G and S thioethylated monomers from arylglycerol-β-ether-linked H, G and S units. In addition, it allows the identification of 5-OH G monomers as observed in maize *bm3* mutants [54]. Among the various mutants analyzed (data not shown), three lines (*Bd4142*, *Bd4604*, and *Bd5139*), released 5-OH G thioacidolysis monomers in higher amounts, compared to the trace amounts detected in the control lines (Table 5). Together with the increased frequency of 5-OH G thioacidolysis monomers, we found that S monomer levels were substantially reduced (by 30 to 40% of the control value). In contrast, H monomers were obtained as minor components (3–6% range) and no substantial difference between mutants and controls was detected. Based on the S levels or S/G thioacidolysis ratio suggests, we hypothesize that COMT activity is lower in *Bd5139* and *Bd4142* compared to *Bd4604*. This hypothesis was further supported by the lignin level of the extractive-free stems as measured by the Klason method. Compared to the corresponding control, this level was reduced by 15%, 10% and 0% in *Bd5139*, *Bd4142* and *Bd4604* respectively (Figure 4).

**Table 5:** Relative frequency (% molar) of *p*-hydroxyphenyl H. guaiacyl G. syringyl S and 5-hydroxyguaiacyl 5-OH G monomers released by thioacidolysis of mature and extractive-free stems from control (Ctrl) and mutant *Bd4142*, *Bd4604*, *Bd5139* lines.

Culture	Sample (n^a^)	% H	% G	% S	% 5-OH G	S/G molar ratio
1	Azygous Control (4)	5.9 (0.3)	37.9 (1.9)	55.7 (1.0)	0.5 (0.0)	1.47 (0.15)
	*Bd4142* (4)	6.2 (0.5)	46.5 (3.1)*	45.3 (3.3)*	2.0 (0.3)*	0.98 (0.14)*
2	Wild-type Control (2)	3.5 (0.3)	30.8 (2.3)	65.4 (2.7)	0.2 (0.0)	2.14 (0.25)
	*Bd4604* (2)	3.2 (0.0)	38.4 (0.3)*	55.1 (3.3)*	3.3 (0.3)*	1.44 (0.02)*
3	Wild-type Control (5)	3.5 (0.5)	31.4 (2.0)	64.8 (2.4)	0.3 (0.1)	2.08 (0.22)
	*Bd5139* (5)	4.0 (0.6)	42.4 (0.4)*	49.5 (1.4)*	4.1 (1.5)*	1.17 (0.04)*

Ctrl (azygous line for *Bd4142* and wild-type line for *Bd4604* and *Bd5139*) and corresponding mutant samples were recovered from plants grown together and in identical conditions.

a number of replicates. The data represent the means and SD (between brackets). Asterisks indicate significant differences compared to the corresponding Ctrl (ANOVA, value at P<0.01).

**Figure 5:**
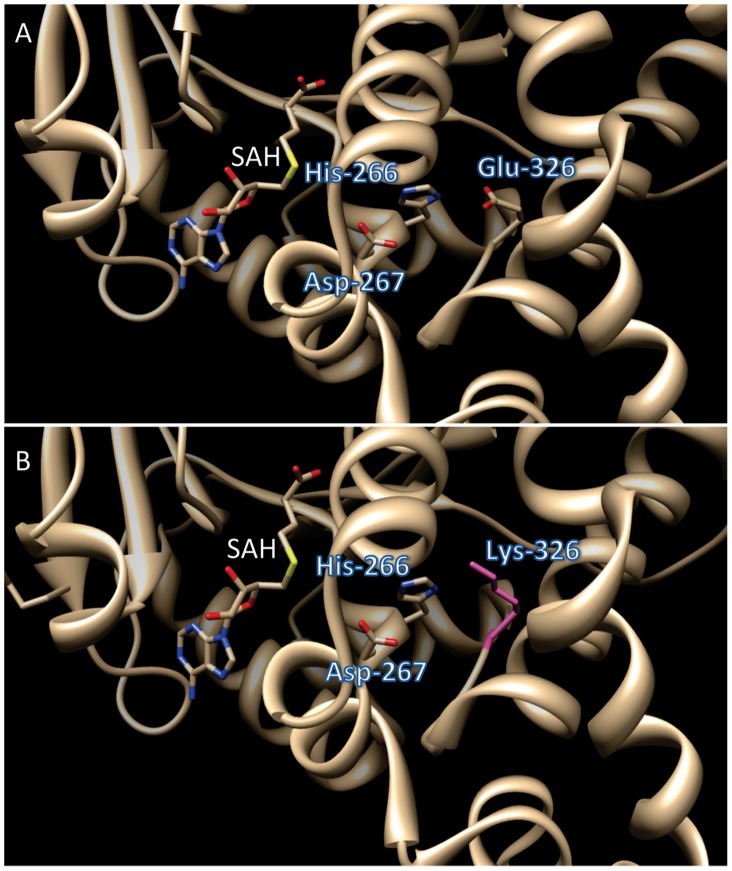
Structural representation of the amino acid substitution of BdCOMT6 protein in the line *Bd4142*. A. 3-D model of wild-type protein highlighting three amino acids important for proper enzymatic activities. B. 3-D model of the protein in *Bd4142* line in which Glu-326 is substitued by Lys-326. SAH, S-Adenosyl-L-homocysteine is shown to illustrate the proximity of the substitued amino acid in the substrat binding pocket.

To further investigate how amino acid substitutions may affect BdCOMT6 activity, we used the 3-D structure, determined for the closely related (90% amino acid sequence identity) *Lolium perenne* OMT, LpOMT [24] to compare WT with mutant proteins. Both COMT enzymes belong to the plant type-1 family of SAM-dependent *O*-methyltransferases, have 360 amino acid residues and possess an auxiliary N-terminal domain that may functions in homodimerization [55], [24]. The *Bd4142* mutation induces a Glu-326-Lys substitution (Figure 5). Glu-326 is thought to be one of the catalytic bases that activate the hydroxyl group of the substrate/ligand [24], whereby Glu-326 provides the hydrogen bond acceptor in an interaction with the His-266 and contributes indirectly to the deprotonation of the phenolic substrate [24]. The Glu-326-Lys substitution, inverts the charge of the aminoacid and therefore is expected to alter proper protein activity and explain the severe reduction of S units detected in this line (Table 5).

The substitution observed in lines *Bd5139* and *Bd7549* is a glycine to aspartic acid at position 256, which is part of a loop facing a β-sheet carrying an amino acid involved in the substrate binding. It is worth noting that two residues (Phe-250 and Asp-248) in the vicinity of Gly-256 are also placed in this loop and are essential for stabilisation of the ligand (SAM/SAH). We speculate that the size and the charge modifications in this mutant could impact the β-sheet and destabilize the SAM binding site, disturbing its function as methyl donor and in consequence reduce the activity of the protein.

Finally, *Bd4604* carries a Gly-246-Asp substitution. This residue is located at the periphery of the protein but still in the SAM/SAH binding domain and adjacent to a α-helical layer and a β-sheet both involved in SAM/SAH binding site conformation. The lateral chain of the aspartic acid in the mutant may turn towards residues involved in the binding of the cofactor. The size and charge differences may modify this site thus hampering the binding of SAH and the activity of the protein.

In conclusion, the three mutant lines that have an altered lignin content and/or composition share at least a mutation located in the vicinity of the SAM/SAH binding and catalytic domain. It is worth noting that the three COMT mutants do not show coloured leaf veins as observed in the brown-midrib mutants of maize, pearl millet or sorghum [56]. In addition, the levels of S units is still high in the mutants (as revealed by the 45 to 55% of S thioacidolysis monomers). This result suggests that enzymatic methoxylation at C5 of the phenolic ring of monolignol is still present in these lines. It remains to be shown whether it is due to residual enzymatic activity of the mutated proteins as shown in a similar allelic series in sorghum [57]. Finally, no grass COMT-deficient mutants or transgenic lines described so far are completely devoid of S units, in contrast to what has been reported in dicots, for example in the Arabidopsis *Atcomt1* mutant [52]. This observation suggests that an alternative pathway may produce S units in grasses.

### Conclusion

We have generated a large collection of chemically-induced mutants useful for forward genetics in *B. distachyon*. A subset of the phenotypes can be consulted at http://urgv.evry.inra.fr/UTILLdb. In addition, BRACHYTIL provides and efficient platform for reverse genetics. This study illustrates the power of this approach by the isolation of an allelic series for BdCOMT6 involved in monolignol biosynthesis. Next generation sequencing techniques will now greatly accelerate reverse genetics approaches in this collection.

## Supporting Information

Information S1 **Amino acid sequences of putative COMT proteins used for phylogenetic analysis in** **Figure 3** **.**
(DOCX)
