# Supplementary material for: A TILLING Platform for Functional Genomics in Brachypodium distachyon
Source: PLoS One. 2013 Jun 19;8(6):e65503. doi: 10.1371/journal.pone.0065503 (PMC3686759; doi:10.1371/journal.pone.0065503)
Supplement: Information S1 — Amino acid sequences of putative COMT proteins used for phylogenetic analysis in Figure 3 . (DOCX) [file pone.0065503.s001.docx]

# Supporting Information S1

>BdCOMT6 Bradi3g16530.1_BRADI

MGSTAADMAATADEEACMFALQLASSSILPMTLKNAIELGLLDTLVQASGKSLTPAEVAAKLPSSSNPAAPDMVDRMLRLLASYGVVSCAVEEGENGKLSRRYAAAPVCKWLTPNEDGVSMAALALMNQDKVLMESWYYLKDAVLDGGIPFNKAYGMSAFEYHGTDPRFNRVFNEGMKNHSIIITKKLLDLYPGFEGLGTLVDVGGGVGATVGAIVARHPAIKGINFDLPHVISEGIPFPGVTHVGGDMFQKVPSGDAILMKWILHDWSDAHCATLLKNCYDALPAHGKVVIVECILPVNPEATPKAQGVFHVDMIMLAHNPGGKERYEREFEELARGAGFTGVKATYIYANAWAIEFTK

>BdCOMT1 Bradi1g14870.1_BRADI

MVVPGAAGAGDEEACMYALQLAASSILPMTLKNAIELGMLEILVAAGKTLSPSQVAERLQAKPGPDAPAMLDRMLRLLASYNVVSCEVEEGQEGLLARRYGPAPVCKWLTPNDDGVSMDPLALLIQDKVSMESWYHLKDVVLDGGLPFNKAHGIIAFEYHGKDARFDRVFNEAMKNHSTILTKKFLEFYTGFDDVKTLVDVGGGVGATIRAIISKYPHISGVNFDLPHVISSAPTCPGVQHIGGDMFKKVPSGDAILMKWILHDWTDDHCMMLLRNCYDALPVGGKLIIIESILPVNPEATPRARMAFEDDMIMLTYTPGGKERYKREFEVLAKGARFASVRTTYIYANSWAIEYTK

>BdCOMT3 Bradi2g02390.1_BRADI

MAEEEAGCMHALMLASSVVQPMAVRTAIELGLLEILVAGAGYGKTMSPEEVTAKLPTSNPEAASMVDRLLRVLASYSVVSCVVEEAKDGSLSRRYGPAPVCKWLTPNEDGVSMAPFCLLAQDRVFTETWCYMKEAILEGRGGAFNKAFGTTWFEHAGVDTRFNNLFNEAMKQHSVIITKKLLELYKGFEGISVLVDVAGGVGATTHAITSRYPSIKGINFDLPHVVAEAPAYPGGRVQHVGGDMFEKVPSGDAIFMKWILNCFSDKDCATLLKNCYDALPAHGKVINLECIMPVNPEPTHGAQGLISVDVSLLAYSPGGKERYLRELEKLAKGAGFADVKATYIYADFWAIEYTK

>BdCOMT2 Bradi2g02380.1_BRADI

MAEEEACMYALQLAVSSVLPMTLKTVIELGILETLVSAGREAPLTPEDLAAKLPAKANPEAASMVDRMLRVLASFNVVSCVVEEAKDGSLSRRYGPAPVCKWLTPNEDGVSMAAFALAAQDKVHMATWPYMKDAVLEGGDPFTKALGMSWFEYAGADTRFNRMYNEAMTHHSGIITKKFLELYTGLDGIGTLIDVGGGIGATIHAVTSKYPTIKGINYDLPHVIADAPAYPGGRVQHVGGNMFEKVPSGADAILMKWILNCFRDEECATLLKNCYDALPAHGKVINVECILPVNPDETPSARGLIQIDMSLLAYSPGGKERYLRELEKLAKGAGFAAVKATYIYANFWAIEYTK

>BdCOMT7 Bradi3g55890

MSAAASEDEELSPAEARLAMMELANMISVPMALTAAIRLGVPGAIWADGANAPLSAADLLPADHPDPSVLERVLRLLASRGVFSEHHGPGPAEPTRRFALTAVGRTLVPAGPSGASYADYVLQHHQDALVLAWPRLHEALLDPAGPEPFARAHRGLPAYAFYAQDKEANEVMLRGMTGVSEPFMEALLDGYAGGFEDVRTLVDVGGSSGACLDMIMRRVGTIAQGINFDLPDVVAAAPPIAGVRHVGGDMFKSIPSGDAIFMKWVLTTWTNDECTAILKNCYGALPEGGKLIACEPVVPETTDTSTRTRALLENDIFVMTTYRTQGRERSEEEFRQLGLAAGFTAFRAIYLDPFYAVLEYLK

>BdCOMT4 Bradi2g19830

MSSLYIYRSTWHVSNQPTTKQSSTKMAPTEVKHSSQDLLQAQVDLWHHALGFVKSMALKCAMELQIPNTI QHHGGAMTPSELATKIGLHPSKLPRLRRLMRVLTVSGIFVVHEAASADKEAVYGLTPTTCLLVSDEVKSNLFPIVTLMLDSTVITPFFGMHSWFLDEHSVSMFKKAHGVTFWEMADQDNTYNQLINNAMVSDSNFLMDIILRECGDVFVGINSLIDVAGGHGGAARAIAKAFPQMKCTVLDLPHVVANAPSDEHVPFISGDMFEYIPPANALFLKWVFHDWGDEDCVKILKKCKEAIPPRDAGGKVIIVDMVVGSGPDEIVTRETQVFFDLFIMYLEGIEREEFEWKKIF MEAGFTDYKIISVLGVRSVIELYP

>BdCOMT8 Bradi4g20020

MAQTTQTTKELESGAELLQAQADLWRHSLGFYTSMALQCAVKLGVPSAIRRSHGATASLPDILDDLSVPPSKLPFLRRVMRLLVTSGVFTSHADETDPSVVYYGLTPVSRLLVDGTVPGSEAVGGRTSQASFVLACTARLNIDAAQGLVGWLQQKPEEEETKPLFSPFAWAHDGASLFERGRVDPEFNGVLNEGMAANSRLGILTVLRECRPLFENLQSLTDCGGGDGATARAITRTFPHVKCTVLDLPHVIAAATVPSDDGIQYVAGDMFESIPPSQAILVKYVLHDWSDEQCVKVLARCREAIPCREAGGKVIVVEVVLGASSPCCAGPMHEAELLMDMAMMCMTTGHEREEHEWRSIFVAAGFSDYKINKALGVQCVIEVYP

>BdCOMT5 Bradi2g19850

MAVTGEEEDKELTMGTEDMLQGHAELCTHAFAYVRSMALKCAVELGIPDAIHRSQCGAATLGELAAMVALPPSRLPRLRRLMRVLAVSNFFTVDDTQQPDGPVYGLTRASRLLVTPPGSGSLSRLVSLMCDPNLAAPFFGMSAWFLTDDDDLRPARSSIFEMHHGADLWDMAARDPALSKSIGDGMDSDSRFIAEVLLLRTDGGGNHHAREVFDGVTSLVDVGGGTGAIASAVAAAFPHIQCTVLDLPHVVAEAPDDGAVRFVAGDMFEDVPPADAVLLKSVMHDWKDDECVRILRRCKEAIPTREAGGKVIIINMVVGSGKAGGEAMLEEAQVVYDLFLMVFEGREREEHEWEKIFLEAGFSGYKVMPVLGIRSIIEVYP

>AtOMT9 At1g21100.1_ARATH

MGYLFQETLSSNPKTPIVVDDDNELGLMAVRLANAAAFPMVLKAALELGVFDTLYAAASRTDSFLSPYEIASKLPTTPRNPEAPVLLDRMLRLLASYSMVKCGKALSGKGERVYRAEPICRFFLKDNIQDIGSLASQVIVNFDSVFLNTWAQLKDVVLEGGDAFGRAHGGMKLFDYMGTDERFSKLFNQTGFTIAVVKKALEVYEGFKGVKVLVDVGGGVGNTLGVVTSKYPNIKGINFDLTCALAQAPSYPGVEHVAGDMFVDVPTGDAMILKRILHDWTDEDCVKILKNCWKSLPENGKVVVIELVTPDEAENGDINANIAFDMDMLMFTQCSGGKERSRAEFEALAAASGFTHCKFVCQAYHCWIIEFCK

>AtOMT15 At1g62900

MRVFELIGSNEQFAEMFNRTMSEASTLIMKKVLEVYKGFEDVNTLVDVGGGIGTIIGQVTSKYPHIKGINFDLASVLAHAPFNKGVEHVSGDMFKEIPKGDAIFMKWILHDWTDEDCVKILKNYWKSLPEKGKVIIVEVVTPEEPKINDISSNIVFGMDMLMLAVSSGGKERSLSQFETLASDSGFLRCEIICHAFSYSVIELHK

>AtOMT16 At3g62000

MSTGLALNRCSVSVCRTAVTLLNRPTVSVARSLKFSRRLIGNCSIAPADPYVVADDDKYGNKQVISLTPRLYDYVLSNVREPKILRQLREETSKMRGSQMQVSPDQAQLLAMLVQMLAAERCIEVGVYTGYSSLAVALVLPESGCLVACERDSNSLEVAKRYYELAGVSHKVNVKQGLAAESLKSMIQNGEGASYDFAFVDADKRMYQDYFELLLQLVRVGGVIVMDNVLWHGRVSDPMVNDAKTISIRNFNKKLMDDKRVSISMVSTDRRWHDDMPQEIVIRKNQLGHSEPDPREKSFKHERISMKPRYTCFRIGNHFFCSESILTNTSKTHLLGMVTTNACTFVKLFNSF

>AtOMT13 At1g21120.1_ARATH

MGYLFEETLSSNPKTPIVVDDDNELGLMAVRLANAAAFPMVLKASLELGVFDTLYAEASRTDSFLSPSEIASKLPTTPRNPGAPVLLDRMLRLLASYSMVKCEKVSVGKGERVYRAEPICRFFLKNNIQDIGSLASQVIVNFDSVFLNTWAQLKDVVLEGGDAFGRAHGGMKLFDYMGTDERFSKLFNQTGFTIAVVKKALEVYQGFKGVNVLVDVGGGVGNTLGVVTSKYPNIKGINFDLTCALAQAPSYPGVEHVAGDMFVDVPTGDAMILKRILHDWTDEDCVKILKNCWKSLPENGKVVVIELVTPDEAENGDINANIAFDMDMLMFTQCSGGKERSRAEFEALAAASCFTHCKFVCQAYHCWIIEFCK

>AtOMT14 At1g21130.1_ARATH

MGYLLEETLSSNSKTPIVIDDDNELGLMAVRLANAAAFPMVLKAALELGVFDTLYAEASRSDSFLSPSEIASKLPTTPRNPEAPVLLDRMLRLLASYSVVKCGKVSEGKGERVYRAEPICRFFLKDNIQDIGSLASQVIVNFDSVFLNTWAQLKDVVLEGGDAFGRAHGGMKLFDYMGTDERFSKLFNQTGFTIAVVKKALEVYQGFKGVNVLVDVGGGVGNTLGVVASKYPNIKGINFDLTCALAQAPSYPGVEHVAGDMFVDVPTGDAMILKRILHDWTDEDCVKILKNCWKSLPESGKVVVIELVTPDEAENGDINANIAFDMDMLMFTQCSGGKERSRAEFEALAAASGFTHCKFVCQAYHCWIIEFCK

>AtOMT12 At1g33030.1_ARATH

MEEQNLSSYAMILSSSSVLPMVLKTAIDLGLFDILAESGPSSASQIFSLLSNETKKHHDSSLVNRILRFLASYSILTCSVSTEHGEPFAIYGLAPVAKYFTKNQNGGGSLAPMVNLFQDKVVTDMWYNLKDSVLEGGLPFNNTHGSSAVELVGSDSRFREVFQSSMKGFNEVFIEEFLKNYNGFDGVKSLVDVGGGDGSLLSRIISKHTHIIKAINFDLPTVINTSLPSPGIEHVAGDMFTNTPKGEAIFMKWMLHSWDDDHCVKILSNCYQSLPSNGKVIVVDMVIPEFPGDTLLDRSLFQFELFMMNMNPSGKERTKKEFEILARLAGFSNVQVPFTSLCFSVLEFHKNK

>AtOMT3 At1g51990.1_ARATH

MISLQTSGGSSEEEDMLLAIQLGGLNFVPYIVKTARELDLFEIMAKARPLGSYLSPVDLASMAAPKNPHAPMMIDRLLRFLVAYSVCTCKLVKDEEGRESRAYGLGKVGKKLIKDEDGFSIAPYVLAGCTKAKGGVWSYLTEAIQEGGASAWERANEALIFEYMKKNENLKKIFNESMTNHTSIVMKKILENYIGFEGVSDFVDVGGSLGSNLAQILSKYPHIKGINFDLPHIVKEAPQIHGVEHIGGDMFDEIPRGEVILMKWILHDWNDEKCVEILKNCKKALPETGRIIVIEMIVPREVSETDLATKNSLSADLTMMSLTSGGKERTKKEFEDLAKEAGFKLPKIIYGAYSYWIIELYPN

>AtOMT4 At1g63140.2_ARATH

MENHLQHSLTIIPKPDLIKEEQRYHEDTVSLQAERILHAMTFPMVLKTALELGVIDMITSVDDGVWLSPSEIALGLPTKPTNPEAPVLLDRMLVLLASHSILKYRTVETGDNIGSRKTERVYAAEPVCTFFLNRGDGLGSLATLFMVLQGEVCMKPWEHLKDMILEGKDAFTSAHGMRFFELIGSNEQFAEMFNRAMSEASTLIMKKVLEVYKGFEDVNTLVDVGGGIGTIIGQVTSKYPHIKGINFDLASVLAHAPFNKGVEHVSGDMFKEIPKGDAIFMKWILHDWTDEDCVKILKNYWKSLPEKGKVIIVEVVTPEEPKINDISSNIVFGMDMLMLAVSSGGKERSLSQFETLASDSGFLRCEIICHAFSYSVIELHK

>AtOMT7 At1g76790.1_ARATH

MGHLIPQTGDEETELGLAAVRLANCAAFPMVFKAAIELGVIDTLYLAARDDVTGSSSFLTPSEIAIRLPTKPSNPEAPALLDRILRLLASYSMVKCQIIDGNRVYKAEPICRYFLKDNVDEELGTLASQLIVTLDTVFLNTWGELKNVVLEGGVAFGRANGGLKLFDYISKDERLSKLFNRTGFSVAVLKKILQVYSGFEGVNVLVDVGGGVGDTLGFVTSKYPNIKGINFDLTCALTQAPSYPNVEHVAGDMFVDVPKGDAILLKRILHDWTDEDCEKILKNCWKALPENGKVIVMEVVTPDEADNRDVISNIAFDMDLLMLTQLSGGKERSRAEYVAMAANSGFPRCNFVCSAYHLWVIELTKQA

>AtOMT5 At1g77520.1_ARATH

MTNHLQDPLPTYPKPVLTKEEQEVDEKMVSLQAESIVNTVAFPMVLKAAFELGVIDTIAAAGNDTWLSPCEIACSLPTKPTNPEAPVLLDRMLSLLVSHSILKCRMIETGENGRTGKIERVYAAEPVCKYFLRDSDGTGSLVPLFMLLHTQVFFKTWTNLKDVILEGRDAFNSAHGMKIFEYINSDQPFAELFNRAMSEPSTMIMKKVLDVYRGFEDVNTLVDVGGGNGTVLGLVTSKYPHIKGVNFDLAQVLTQAPFYPGVEHVSGDMFVEVPKGDAVFMKWILHDWGDEDCIKILKNCWKSLPEKGKIIIVEFVTPKEPKGGDLSSNTVFAMDLLMLTQCSGGKERSLSQFENLAFASGFLRCEIICLAYSYSVIEFHK

>AtOMT6 At1g77530.1_ARATH

MSNHLQDPLTTYPKPGLTKEEQEIDEKMVSLQAESIVNAVAFPMVLKAALELGVIDTIAAASNGTWLSPSEIAVSLPNKPTNPEAPVLLDRMLRLLVSHSILKCCMVESRENGQTGKIERVYAAEPICKYFLKDSDGSGSLSSLLLLLHSQVILKTWTNLKDVILEGKDAFSSAHDMRLFEYISSDDQFSKLFHRAMSESSTMVMKKVLEEYRGFEDVNTLVDVGGGIGTILGLITSKYPHIKGVNFDLAQVLTQAPFYPGVKHVSGDMFIEVPKGDAIFMKWILHDWGDEDCIKILKNCWKSLPEKGKVIIVEMITPMEPKPNDFSCNTVLGMDLLMLTQCSGGKERSLSQFENLAFASGFLLCEIICLSYSYSVIEFHK

>AtOMT8 At3g53140.1_ARATH

MENESSESRNRARLAIMELANMISVPMSLNAAVRLGIADAIWNGGANSPLSAAEILPRLHLPSHTTIGGDPENLQRILRMLTSYGVFSEHLVGSIERKYSLTDVGKTLVTDSGGLSYAAYVLQHHQEALMRAWPLVHTAVVEPETEPYVKANGEAAYAQYGKSEEMNGLMQKAMSGVSVPFMKAILDGYDGFKSVDILVDVGGSAGDCLRMILQQFPNVREGINFDLPEVVAKAPNIPGVTHVGGDMFQSVPSADAIFMKWVLTTWTDEECKQIMKNCYNALPVGGKLIACEPVLPKETDESHRTRALLEGDIFVMTIYRTKGKHRTEEEFIELGLSAGFPTFRPFYIDYFYTILEFQK

>AtOMT2 At4g35150.1_ARATH

MEESKRNLLDEEAKASLDIWRYVFGFADIAAAKCAIDLKIPEAIENHPSSQPVTLSELSSAVSASPSHLRRIMRFLVHQGLFKEVPTKDGLATGYTNTPLSRRMMITKLHGKDLWAFAQDNLCHSQLINEAMACDARRVVPRVAGACQGLFDGVATVVDVGGGTGETMGILVKEFPWIKGFNFDLPHVIEVAQVLDGVENVEGDMFDSIPASDAVIIKWVLHDWGDKDCIKILKNCKEAVLPNIGKVLIVECVIGEKKNTMIAEERDDKLEHVRLQLDMVMMVHTSTGKERTLKEWDFVLTEAGFARYEVRDFDDVQSLIIAYRS

>AtOMT17 At4g35160.1_ARATH

MSSDQLSKFLDRNKMEDNKRKVLDEEAKASLDIWKYVFGFADIAAAKCAIDLKIPEAIENHPSSQPVTLAELSSAVSASPSHLRRIMRFLVHQGIFKEIPTKDGLATGYVNTPLSRRLMITRRDGKSLAPFVLFETTPEMLAPWLRLSSVVSSPVNGSTPPPFDAVHGKDVWSFAQDNPFLSDMINEAMACDARRVVPRVAGACHGLFDGVTTMVDVGGGTGETMGMLVKEFPWIKGFNFDLPHVIEVAEVLDGVENVEGDMFDSIPACDAIFIKWVLHDWGDKDCIKILKNCKEAVPPNIGKVLIVESVIGENKKTMIVDERDEKLEHVRLMLDMVMMAHTSTGKERTLKEWDFVLKEAGFARYEVRDIDDVQSLIIAYRS

>AtOMT10 At5g37170.1_ARATH

MTNHHQESLTTYPKPGPTREQEQVDEEMMSMQMQALRITNSLAFPMGVWLSPSEIAFGLPTKPTNPEAPMLIDRMLRLLVSHSILKCRLVETGENNRTESTQRVYAAEPDTSEGCDTRRKRCIQFCPWHGTLRIRCTDEQFAAIFNQAMSDSSTMIMTKILEVYKGLKDVNTLVDIGGGLGTILNLVISSKYPQIKGINFDLAAVLATAPSYPGVEHVPGDMFIDVPKGDAIFMRRILRDWNDKDCVKILTNCWKSLPEKGKVIIVDMVAPSEPKSDDIFSKVVFGTDMLMLTQCSCGKVRSFAQFEALASASGFHKCEVSGLAYTYSVIEFHK

>AtOMT11 At5g53810.1_ARATH

MANHLQVPLTKPDRVKEEQEVEEEARLLARRLANAAASPMVLKAALELGVIDTITTVGGGDLWLSPSEIALRLPTKPCNLEAPALLDRMLRFLVSHSVLKCRTVIEENGQTGKVERVYAAEPVCKYLLNKSDDVSGSFASLFMLDLSDVFIKTWTHLEDVILEGRDAFSSAHGMKLFEYIQADERFGKVFNRAMLESSTMVTEKVLKFYEGFKDVKTLVDVGGGLGNTLGLITSKYPHLIGINFDLAPVLANAHSYPGVNHVAGDMFIKIPKGDAIFMKWILHDWTDEQCVAILKNCWKSLEENGKLIIVEMVTPVEAKSGDICSNIVFGMDMTMLTQCSGGKERDLYEFENLAYASGFSRCAIVCAVYPFSVIEIYK

>AtOMT1 At5g54160.1_ARATH

MGSTAETQLTPVQVTDDEAALFAMQLASASVLPMALKSALELDLLEIMAKNGSPMSPTEIASKLPTKNPEAPVMLDRILRLLTSYSVLTCSNRKLSGDGVERIYGLGPVCKYLTKNEDGVSIAALCLMNQDKVLMESWYHLKDAILDGGIPFNKAYGMSAFEYHGTDPRFNKVFNNGMSNHSTITMKKILETYKGFEGLTSLVDVGGGIGATLKMIVSKYPNLKGINFDLPHVIEDAPSHPGIEHVGGDMFVSVPKGDAIFMKWICHDWSDEHCVKFLKNCYESLPEDGKVILAECILPETPDSSLSTKQVVHVDCIMLAHNPGGKERTEKEFEALAKASGFKGIKVVCDAFGVNLIELLKKL

>OsOMT2 Os02g57760.1_ORYSA

MGGGGDGELSPAEARLAMMELANMISVPMALTAVIRLGVPAKLWAGGANAPLAAADLLPAGHPDPSVLERLLRLLASRGVFSEHTGSSSPSPRRFSLTAVGRTLVPGGGGSPSGSGASYADYVLQHHQDALVRAWPLLHEAVLDPSGPEPFARANAGVPAYAYYGKDREANEVMLRAMTGVSEPFMEALLEGYGDGGFEGVSTLVDVGGSSGACLEMIMRRVRTIRDGVNFDLPDVVAAAPPIPGVRHVGGDMFKSIPSGDAIFMKWVLTTWTNEECTAILSNCHKALPGGGKVIACEPVVPDTTDGSTRTRALLENDIFVMATYRTQGRERSEEEFRHLGLAAGFASFRAIYLDPFYAVLEYTK

>OsOMT3 Os04g01470.1_ORYSA

MDKMTPAADGDDDETTCIRALELIFTFVVPMTLKATIKLGLLDALTGGGHALTADELAAAAQLPAEAASSVDRMLRLLASLDVVKCAPTDTGGEAAVRRYTPAPVCRWFAGERSLAPLAMFLLDDDYLSTWNQLPAAVAGGDGQVAFEKARGMPMFEYMGTNRRLNTLFNQAMVQQSTVVIGKLLERFQGFDGVSVLVDVGGGTGATLEMITSRYKNITGVNFDLPHVIAQAPSLPGVKHIAGNMFESVPNGDAIFLKSMLHLHNDEDCIKILKKCHQALTHNGKVIAVEILLPAIPEPVPTAQNPFRMDMIMLNNHWGGKERTEPEFAKLAVECGYTGVFQATYIFANYWALEFSK

>OsOMT4 Os04g09604.1_ORYSA

MDPYTSRAPASGGVAAGDDDEEAACLQAFELMCIFTVPMTLKAAIELGLLDALAAAGDGRALTADELAAARLPDAAPDKAEAASSVDRMLRLLASFDVVKCSTEAGPGGEPPRRRYSPAPVCRLFTAGGNSHRGSLAPSVLFGVDEDYLCTWRQLAAAVGGGGPSAFERAHGMRMFEYMGTNRRLNTLFNQAMAQQSMIVIDKLLDRFHGFDGVGVLVDVGGGTGATLEMITSRYKHITGVNFDLPHVISQAPSIPGVKHIAGNMFESISNIGDAIFLKMILHMQNDEDCIKILKNCHQALPDNGKVIAVEIVLPTIPDLAQTARYPFQMDMIMLSNSRGGKERTELEFAKLATDSGFSGALRTTYILANYWVLEFSK

>OsOMT5 Os04g09654.1_ORYSA

MASGISRTPATGVTAGGGDDEEAAWLHALELISGFTVSMTLKAAIQLGLIDALTAAADGRALTAGELVAQLPAVDDAEAATSVDRMLRLLASFNVVRCSTEAGPGGDPLRRYSPAPVCRWFTAGDNHQGSLAPRLMLDVDEDNLSTWHQMAAAVVSGGPSAFERAHGMPLFEYMGTNHRFNMLFNQAMSQQSMMVMNKLLDRFHGFDGISVLVDVGGGTGVTLKMIISRYKHITGVNFDLPHVISQAPSLPGVNHVAGNMFESVPKGDAIFLKSMLLRNDEECIKILKNCHYALSDNGKVIVVDIVLPETPKPVPEAQNPLRMDVMMLNNLRGGKIRTEQEYAKLAMDSGFSGSFRTTYIFANFMAIELCK

>OsOMT6 Os04g09670.1_ORYSA

MPPYPCVKHVAGNMFESIPNGDAIFLKSILHLQNDEDCIKILKNCHQALSDNGKLIAVEIVLPAIPEPVPTAQYPFQMDMIMLNNFRGGKERTELEFTKLAMDSSFSGTLRTTYIFANYWALEFNK

>OsOMT7 Os04g09680.1_ORYSA

MLRLLASCNVVKCSTEAGPAGEPLRRRYSPAPVCRWFTAGGNSHHGSLAPSVLFGIDEDYLSTWHQLAAAVGGGGAVAFERAHGAPMFEYMGTNRRLNTLFNQAMAQQSMIVINKLLDRFHGFDGVGVLVDVGGGTGGTLEMIMSRHKHITGVNFDLPHVISQAPSLPGYV

>OsOMT1 Os08g06100.1_ORYSA

MGSTAADMAAAADEEACMYALQLASSSILPMTLKNAIELGLLETLQSAAVAGGGGKAALLTPAEVADKLPSKANPAAADMVDRMLRLLASYNVVRCEMEEGADGKLSRRYAAAPVCKWLTPNEDGVSMAALALMNQDKVLMESWYYLKDAVLDGGIPFNKAYGMTAFEYHGTDARFNRVFNEGMKNHSVIITKKLLDLYTGFDAASTVVDVGGGVGATVAAVVSRHPHIRGINYDLPHVISEAPPFPGVEHVGGDMFASVPRGGDAILMKWILHDWSDEHCARLLKNCYDALPEHGKVVVVECVLPESSDATAREQGVFHVDMIMLAHNPGGKERYEREFRELARAAGFTGFKATYIYANAWAIEFTK

>OsOMT8 Os12g09770.1_ORYSA

MDNARESEDEHCLYAQELVFAYNRSMVLRAAIQLGLLDALAAGGDALTTDELAGKIQATDGVAVDRILRFLASFDVVRCSTETSPDGGAALIRRYTPAPVCRWLTKNNGEGSLAPFSMFIIDEDHLLPWQHIAEAVASGGPAPSERTHGMPYHEYIGKNKRLGGLFDHAMAQHSAIRARKMLERFEGFDGIQRLVDAGVEHIAGDMYESVPNGDAILLQWMLLMFSDEDCIKILKNCHQALPEGGKVIIVEGLLPETPNTTPAAQDSFTMDMILFVLFKVGKHRTEQEFAKLAKESGFTGFTAGLNVGMDSESNTIKSARRSPRIKSSSKSQSNTQYPTLIFVLPMTVVPLYGGGRIDREDDHIGEKNVMAFTAPPPTCAAVVEVVATCACLPPHLNARLLVTGRERKRVGAPAREEERRALRLDDMEEAEDPTTKLKTTLEGGDASGDGES

>OsOMT9 Os12g10140.1_ORYSA

MEKIVAASDAQEKEDEHCLYAQELMFAYNRSMVLRAAVQLGLLDALSAAAGNALTADELAEKIQATDKAEVAVSVDRILRYLASFDVVRCSTETSPDGALLRRYMPAPVCRWLTRNNGEGSLAPFTVFVVDEDHLLPWQHIAAAVTSGGPAPFERAHGLLYFEYMGKNQRLGALFDHAMAQHSVILVSKMLERFQGFDGVQQLVDVGGGDGSTLGMITSRYKHIRGINYDLPHVISQAPSLPETTIDASN

>OsOMT10 Os12g10170.1_ORYSA

METCVEHIAGNMYESVPNGDAILLQWMLLMFSDEDCIKILKNCHQALPKGGKVIIVDGLL

PETPNTSPAARDSFTMDMIMFVLFKVGKQRTEQEFAKLAKEAGFTGTFRSTYIFLNFYALEFNKQLYT

>OsOMT11 Os12g13800.1_ORYSA

MAHHRVTDDMSSVTGVKYMLNDSDKHRQNNNIGYYLKEAVSEGGTAFDKAYGTSLFQYLGQDGNEPSNTLFNQAMASHSVVITNKLLQFFRGFDAGAGVDVLVDVGGGVGATLRMITARHPHLRGVNYDLPHVIAQAPPVEGVEHIGGSMFDHVPSGSAILLKWILHLWGDEECVKILKNCYKALPAKGKVILVEYVLPASPEATLAAQEAFRLDVMMLNRLAGGKERTQQEFTDLAVDAGFSGDCKPTYIFTNVWALEFTK

>PtCOMT POPTR_0012s00670.1_POPTR

MGSTGETQMTPTQVSDEEAHLFAMQLASASVLPMILKTAIELDLLEIMAKAGPGAFLSTSEIASHLPTKNPDAPVMLDRILRLLASYSILTCSLKDLPDGKVERLYGLAPVCKFLTKNEDGVSVSPLCLMNQDVLMESWYYLKDAILDGGIPFNKAYGMTAFEYHGTDPRFNKVFNKGMSDHSTITMKKLLETYKGFEGLTSLVDVGGGTGAVVNTIVSKYPSIKGINFDLPHVIEDAPSYPGVEHVGGDMFVSVPKADAVFMKWICHDWSDAHCLKFLKNCYDALPENGKVILVECILPVAPDTSLATKGVVHIDVIMLAHNPGGKERTEKEFEGLAKGAGFQGFEVMCCAFNTHVIEFRKN

>FaCOMT AAK68909

MGSTAADMAASADEEACMFALQLASSSILPMTLKNAIELGLLEILVAAGGKSLTPTEVAAKLPSAANPEAPDMVDRMLRLLASYNVVSCLVEEGKDGRLSRSYGAAPVCKFLTPNEDGVSMAALALMNQDKVLMESWYYLKDAVLDGGIPFNKAYGMSAFEYHGTDPRFNRVFNEGMKNHSIIITKKLLELYHGFQGLGTLVDVGGGVGATVAAITAHYPAIKGVNFDLPHVISEAPPFPGVTHVGGDMFKEVPSGDAILMKWILHDWSDQHCATLLKNCYDALPAHGKVVLVECILPVNPEAKPSSQGVFHVDMIMLAHNPGGRERYEREFEALARGAGFTGVKSTYIYANAWAIEFTK

>LpCOMT AAD10253

MGSTAADMAASADEDACMFALQLASSSVLPMTLKNAIELGLLEILVAAGGKSLTPTEVAAKLPSAANPEAPDMVDRILRLLASYNVVTCLVEEGKDGRLSRSYGAAPVCKFLTPNEDGVSMAALALMNQDKVLMESWYYLKDAVLDGGIPFNKAYGMSAFEYHGTDPRFNRVFNEGMKNHSIIITKKLLELYHGFEGLGTLVDVGGGVGATVAAIAAHYPTIKGVNFDLPHVISEAPQFPGVTHVGGDMFKEVPSGDTILMKWILHDWSDQHCATLLKNCYDALPAHGKVVLVQCILPVNPEANPSSQGVFHVDMIMLAHNPGGRERYEREFQALARGAGFTGVKSTYIYANAWAIEFTK

>PvCOMT ADX98508

MGSTATDVAAAADEEACMYALQLASSSILPMTLKNAIELGLLEVLQKDPAAALAPEEVVAQLPVAPANPDAAAMVDRMLRLLASYDVVRCQMEEGKDGRYSRRYAAAPVCKWLTPNEDGVSMAALALMNQDKVLMESWYYLKDAVLEGGIPFNKAYGMTAFEYHGTDPRFNRVFNEGMKNHSVIITKKLLEFYAGFEGVGTLVDVGGGVGATLHAITSRYPGIRGVNFDLPHVISEAPPFPGVEHVGGDMFKAVPAGDAILMKWILHDWSDAHCAAILKNCYDALPAGGKVIAVECILPVNPEATPKAQGVFHVDMIMLAHNPGGKERYEREFEELAKGAGFTGFKATYIYANAWAIEFTK

>SbCOMT Sb07g003860.1_SORBI

MGSTAEDVAAVADEEACMYAMQLASSSILPMTLKNALELGLLEVLQKDAGKALAAEEVVARLPVAPTNPAAADMVDRMLRLLASYDVVKCQMEDKDGKYERRYSAAPVGKWLTPNEDGVSMAALALMNQDKVLMESWYYLKDAVLDGGIPFNKAYGMTAFEYHGTDPRFNRVFNEGMKNHSVIITKKLLEFYTGFDESVSTLVDVGGGIGATLHAITSHHSHIRGVNFDLPHVISEAPPFPGVQHVGGDMFKSVPAGDAILMKWILHDWSDAHCATLLKNCYDALPEKGGKVIVVECVLPVTTDAVPKAQGVFHVDMIMLAHNPGGRERYEREFRDLAKAAGFSGFKATYIYANAWAIEFIK

>ZmOMT1 AC196475.3_FGP004_ZEAMA

GSTAGDVAAVVDEEACMYAMQLASSSILPMTLKNAIELGLLEVLQKEAGGGKAALAPEEVVARMPAAPGDPAAAAAMVDRMLRLLASYDVVRCQMEDRDGRYERRYSAAPVCKWLTPNEDGVSMAALALMNQDKVLMESWYYLKDAVLDGGIPFNKAYGMTAFEYHGTDSRFNRVFNEGMKNHSVIITKKLLDFYTGFEGVSTLVDVGGGVGATLHAITSRHPHISGVNFDLPHVISEAPPFPGVRHVGGDMFASVPAGDAILMKWILHDWSDAHCATLLKNCYDALPENGKVIVVECVLPVNTEATPKAQGVFHVDMIMLAHNPGGKERYEREFRELAKGAGFSGFKATYIYANAWAIEFIK

>ZmOMT2 GRMZM2G408458

MGHQAQHATDDTEELLAAHRELWCHALGYVKSMALKCALDLRIPDIIQRCGGSATLGQLLAASEVPASNLGYLRRVMRTLTAMRIFAVVGHGHGPDKADDPADDDATAVSYRLTPASRLLATSDDDDDASKNNLSIHPNISSHVRPKNVSLLFSMAEWMKDEQALSVSLYETVNRKCMWACVEDDAATRACFYESMDADTRLVMQAVIRKCPAVFDDGLTSLVDVGGARGTAAAAVVAAFPHIQKCTVMDLPHIVAEAPAGTGLCFHGGDMFEHIPSADAVMLKVYIYTNWYQMDWILHDWDDDKCVKIMERCKEAISGKEGRGGKVIIIETVLGSRPDDDATCKETYVLDLQILSFVNGAEREEHEWRRIFLAAGFRDYKITHTRGIPSIIEVFP

>ZmOMT3 GRMZM2G036048

MASEVVRPSDAELLQAQADIWRLSLSYLTPLSLKCAVVLGIPRAIYRHGGAASAAELITALSLPSAKLPFLRRLLRLLAASGVFTVDRQSTEEERYRISPVSYLLVEGIPHEDHMNHTSFVLTCTSSRYIEPAIGLAEWFRKDTATSPFEELHGATLFHESMGSLDADFHDMASDALDTHDNFGVEMALREFRDLFEGIQSMTYCCGNFGDDKGARAIAKAFPHIKCTVLAPPKIIAAKPADGVKINYVEGDMFSFIPPAQTVVLKLVLHTTGIR

>ZmOMT4 GRMZM2G085924

MASEVVRPSDAELLKAQADIWRLSLSYLTPLSLRCAVELGIPTAIYRHGGAASAAELVTALSLPSTKLPFLRRLLRLLAASGVFTVDKQSSEEERYRISPVSYLLVDGIPHEDHMNHTALVLTCTSTRYIEAGIGLAEWFKRDVVTSPFEELHGATLFHESMGSLDADFHDMASEALDAHDNFGIEIAMREFRDLFEGIQSMTYCCGNFGDDKGARAIVKAFPHIKCTVLAPPKIIATKPADGAMINYVEGDMFSFIPPAQTVVLKLVLHHLTDEECVKLLAQCRKAIPSRKDGGKVIIGDIMIDHSGPMLETHLLMDIGMMTMTKGRQRDEKEWSELFTKAGFSEYKILKEFGARVAFEVYP

>ZmOMT5 GRMZM2G147491

MAALAPSIVVPTDAELLQAQADLWRNSLCYLKSMALKCATELGIPTAIYSLGGAASLPDLIASLSLPQAKLPFLGRLMRLLSSSGVFAVVESPEAVYSLTPLSYLLVDGIAADNNHMDHAPFLLTVTSAHYIDLAIDLADWFKKEAKTPPFDHKHAASLFEESMERKAPGFHKMSILGLLVHDNFATSIAVREYQDVFQGVKSVTDCCYHGDGTTGKALAKAFPLIKITVLDLPQEIRKIPADGVVNYVGGDMFKSIPRAQMVLLKMVLHHWSDEDCVKILANCRKAIPSREEGGKVVIADIILDPASGPVMFQTQLLMDVCMMLMKGGRQRDVNDWRDLIQKAGFSDYKLLKKFGARGVLEIYP

>ZmOMT6 GRMZM2G093092

MALSTQDLLEAHVELWHQSLCYAKSLALAVALDLRIPDAIHHHGGSATLPQILAETAAHPSKLRALRRLMRVLTVSGTFSVEQQPPAGGDDSTVDAPDEAVYRLTAASRFLVSDEVSSSTLAPFVSLALHPIAVSPHTMGICAWFRQEQREPSPYGLAFQQTFPTIWEHADDVNALLNKGMVADSRFLMPIMLRECGEVFRGIESLVDVGGGHGGATAAIAAAFPHLKCSVLDLPHVVAGAPSDVNVQFVAGNMFQSIPPATAVFLKTTLHDWGDDECVKILKNCRQAISPRDAGGKVIILDMVVGYGQPNITHLETQVMFDLYIMTVNGAERDEQEWKKIFIEAGFKDYKILPILGALSVIEVYP

>ZmOMT7 GRMZM2G106172

MAFTEESSQDLLQAHDELWHQSVSYLKSLALTVALDLRIPDAIHHHGGGATLLQILDKTALHQSKLRALRRLMRVLTVSGTFSVVQQPPCGDDDSTVYRLTAASRFLVSEEVSSATLAPFMSVVLHPISQSSHARGICAWFRQEHHDPSAFGLAFGQAPTIWEHADDTNAILNKGLAAQSRFLVPVMLRECGEAVFRGIDSLVDVGGGHGGAATAIAAAFPHLKCSVLDLPHVVAGAPSDGNVQFVAGDMFQSIPPATAVFLKTALHDWGDDECVKILKNCRQAISPCDEGGKVIIMDMVVGYDESNTKRLEVQILFDLFIMMVNGAERDEQEWKKIFIQAGFKDYKILPVVGSLSVIEVYP

>ZmOMT8 GRMZM2G097297

MALSKEQKLTISEQQHTASSEQQVALDAELQLWNHTFGYVKSMALKAALDLGIPDAIHQHGGSATIPQIVTRITLHPSKTPCLRRLMRVLTVTGVFGTQEPHDDGGGCDDELVYTLTPASRLLVGPPGQNVSPLLNVMLCPIFVSSFLDLRGWFQHEMPDPSPFKVTHGRDIWELAAHDAGFSRLFDAGMVADSGFIMNVVVRECGSGTVFQGISSLVDVGGGFGGATQAIAKAFPHLECSVLDLPNVVAGAPADTAVKYVAGDMFESVSSADAVFLKSIIHDWGDADCVKILKNCKKAIPAQGGKVIILDIVVGAGSSSCDRKNVETQCLFDLYIMTINGVERDEREWKKIIFEAGFTSYKIIPVLGTRSIIEVCP

>ZmOMT9 GRMZM2G140996

MALTTSTNNQALLDAQLELWHTSFAFIKSMALKSAVDLGIADAIHSHGGNATLPQIVSRAALHPSKIPCLRRLMRVLTAAGIFSAAHRSPDDGGGELVYGLTPASQLLVGGSSSLAPFMSLVLHQVYVSPFLGLGTWLQHERSDPTLFEMTHGLTAWDLNDHKPAFGELFNQGMVCDSSFVMDIVVKECGDVFRGLSSLVDVAGGLGAAALAISTAFPHVQCSVLDLPHVAANAPANTSVKYIAGDMFESIPPADAVFLKWVLHDWGDADCIKILKNCKKAIPSRDAGGKVIIVDMVVGGQSSNIKHKETQVLFDLFIMTINGAERDEHEWKKIISEAGFSDYKIIPVLGVRSIIEVYP

>ZmOMT10 GRMZM2G141026

MAPAKAQHTSTNQQTLLDAQLQLWHHAFGYVKSMALKAALDLGIPDAIHQHGGSATLPQIVTELAALHPSKTPCLRRLMRVLTLTGVFGVVVQHSTTDGGSDLVYELTPASRLLVGSAPSNGPNVSPFLNMILGTAFVSSFLDLGEWFQHELPDPSPFKLAHGQHVWDMARHDASFAKLCDSGMVADSGFIMDVMVEECADGVFRGISSLVDVAGGLGGAAQAIAKAFPHIDCSVLDLPNVVAAAPTSTDVKYIAGDMFQSIPAADVVFLKWVLHDWGDAECVKILQNCKKAIPSEGGKVIIMDIVVGAGSSDRKHVETQVLFDLFIMAINGAERDEEEWKKIIFEAGFSSYNIIPVLGVRSIIEVYP

>ZmOMT11 GRMZM2G102863 GRMZM2G124799

MAPANVQHTSTNQQTMLDAQLQLWHHTIGYVKSMALKAAVDLRIADAIHQHGGSATLPQIVTKVTLHPSKIPCLRRLMRVLTLTGVFSVHAGGDEPVYGLTPASRLMVSPGPNLTPFLTLLLSTFFVSSFLDLNEWFQHETGPSPFELANGRDIWALSGHDASFGKLFDDGMVADSGFIMEVVVKECGDVFRGVGSLVDVAGGLGGATQTIAKAFPDMECSVLDLSHVVANAPTDTTVKYIAGDMFESIPSANVVFLKWILHDWGDAECVKILKNCKKAIASQEEGKVVILDMVVGAGSSDEKHVEMQIVFDLFMMFINGTERDETEWKKIIFEAGFSRYKIIPVLGVRSIIEVYP

>ZmOMT12 GRMZM2G124799

MTLSKKQGAGTDQQALLDAQLQLWHHTIAFVKSMALKAAVDLRIADAIHLHGGSATLSQIVTKVTLHPSKIPCLRRLMRVLTLTGVFSVNSGAVVDEPVYGLTPASRLLVGPGLNQTPFLTLMLSTFFVSSFLGLDEWFQHETGPSPFELANGRDIWTLSGHDASFGKLFDDGMVADSGFIMDVVVKECGDVFRGVGSLVDVAGGLGGATQTIAKAFPDVACSVLDLSHVVANAPTDTTVKYIAGDMFESIPSANVVFLKWILHDWGDAECVKILKNCKKAIASQEEGKVVILDMVVGAGSSDEKHVETQIVFDLFMMFINGTERDETEWKKIIFEAGFSRYKIIPVLGVRSIIEVYP
